# Interferon therapy for chronic hepatitis B in children: an immunological perspective

**DOI:** 10.3389/fimmu.2026.1778814

**Published:** 2026-03-17

**Authors:** Yinan Zhao, Yige Wang, Guoying Yu

**Affiliations:** 1 Infectious Diseases Major, Qinghai University, Xining, China; 2 Department of Hepatology II, Fourth People’s Hospital of Qinghai Province, Xining, China

**Keywords:** adverse effects, children, chronic hepatitis B, immune modulation, interferon therapy, personalized medicine, therapeutic challenges

## Abstract

Chronic hepatitis B (CHB) remains a significant global health issue, particularly in children, where the virus can lead to long-term hepatic damage and immune system dysregulation. Interferon (IFN) therapy, such as pegylated interferon (PEG-IFN), has served as a fundamental approach in the management of CHB by facilitating immune modulation and viral suppression. Nevertheless, the application of this treatment in children presents unique challenges, including heterogeneous immune responses, potential adverse effects, and constraints regarding long-term efficacy. This perspective discusses the immunological mechanisms associated with IFN therapy in pediatric CHB, emphasizing its potential to enhance immune-mediated clearance and suppress viral activity. We additionally examine the primary clinical challenges, including treatment resistance, adverse effects, and the necessity for personalized approaches to optimize therapeutic outcomes. Furthermore, this study examines prospective developments in IFN therapy, encompassing innovations in drug formulations, combination treatment strategies, and the implementation of personalized medicine approaches. Despite the challenges associated with IFN therapy, it continues to be a promising treatment modality. Furthermore, ongoing research into its combination with other immunomodulatory agents holds potential for developing more effective and sustainable management strategies for children with CHB.

## 1 Introduction

Chronic hepatitis B (CHB) continues a significant global health concern, with approximately 257 million people worldwide living with persistent hepatitis B virus (HBV) infection (1, 2). Annually, chronic HBV infection accounts for approximately 880,000 fatalities, predominantly resulting from complications including decompensated cirrhosis, liver failure, and hepatocellular carcinoma (HCC) (2, 3). Despite ongoing vaccination efforts, which have significantly reduced the incidence of new infections, the challenge of chronic HBV persists (4). The World Health Organization (WHO) has established an objective to globally eradicate HBV infection by the year 2030, with the specific goals of achieving a 90% reduction in HBV incidence and a 65% decrease in associated mortality rates (2, 5). The global infection rate of chronic hepatitis B among children under 5 years old is about 1.3%, and that among adolescents aged 5–19 years old is about 0.8%. Moreover, there are some regional differences in new infection cases among children. The incidence rate is the highest in the Western Pacific and Africa, and perinatal and early childhood transmission accounts for more than 90% of local pediatric cases (6, 7). A large number of children are infected with the virus through mother to child transmission from HBV positive mothers during the perinatal or early childhood period. These children have a high risk of chronicity, with up to 90% of infants infected during the perinatal period developing chronic hepatitis. Some children may gradually progress to severe liver disease (8). Although vaccination significantly reduced the rate of new infection, the World Health Organization still proposed the goal of global eradication of HBV infection by 2030, that is, to reduce the incidence rate and related mortality. The prevention and treatment of high-risk groups such as children became the key. In children, chronic HBV infection often presents as chronic inflammation, increased liver enzymes, and sometimes hepatomegaly, jaundice, or cirrhosis (9). Persistent childhood infections can cause progressive liver damage, potentially resulting in cirrhosis and liver cancer in adulthood (10). Consequently, the implementation of early treatment interventions is of paramount importance.

Interferon (IFN) therapy, especially pegylated interferon (PEG-IFN), has long been used to manage CHB, aiming to enhance the immune system’s ability to suppress the virus (11, 12). However, its application in children presents unique challenges, including issues related to treatment efficacy, side effects, and immune system modulation (11, 13, 14). Despite these challenges, IFN therapy continues to be a pivotal therapeutic option in the treatment of pediatric hepatitis B, providing potential benefits in terms of immune activation and viral suppression (15, 16).

This article aims to address four key questions:

1. How do INF modulate the immune response in children with CHB?
2. What are the clinical challenges and barriers to effective IFN therapy in pediatric populations?
3. How can future research improve the safety and efficacy of IFN treatment for children?
4. What are the potential advancements in combination therapies and personalized treatment strategies for pediatric CHB?

**Figure 1:**
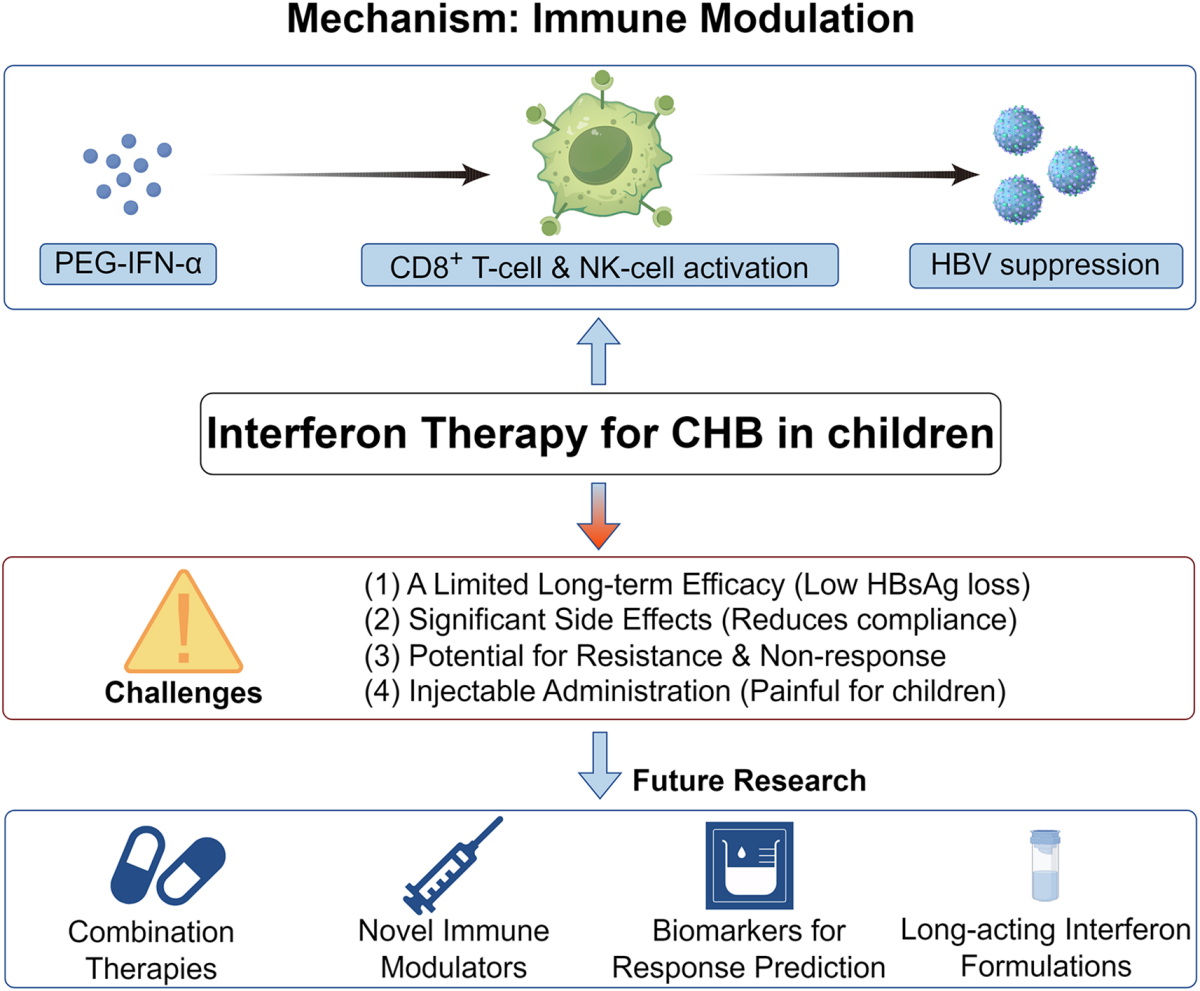
Current immunotherapy strategies of Interferon for CHB, current status, and challenges. This figure presents a three-layer structure for Interferon Therapy for CHB in Children. The top layer illustrates the impact of interferon on immune cells, showing how it enhances immune responses such as activation of T-cells and NK-cells, leading to HBV suppression. The middle layer displays the challenges faced by interferon therapy, such as side effects, resistance, and treatment failure. The bottom layer outlines future research directions, including combination therapies and advancements in interferon formulations.

**Figure 1** is intended to visually depict the principal components of IFN therapy in pediatric patients with CHB. This highlights the significant role and inherent limitations of IFN in the management of CHB among pediatric patients.

## 2 Immunological mechanism of interferon treatment for CHB in children

**Figure 2:**
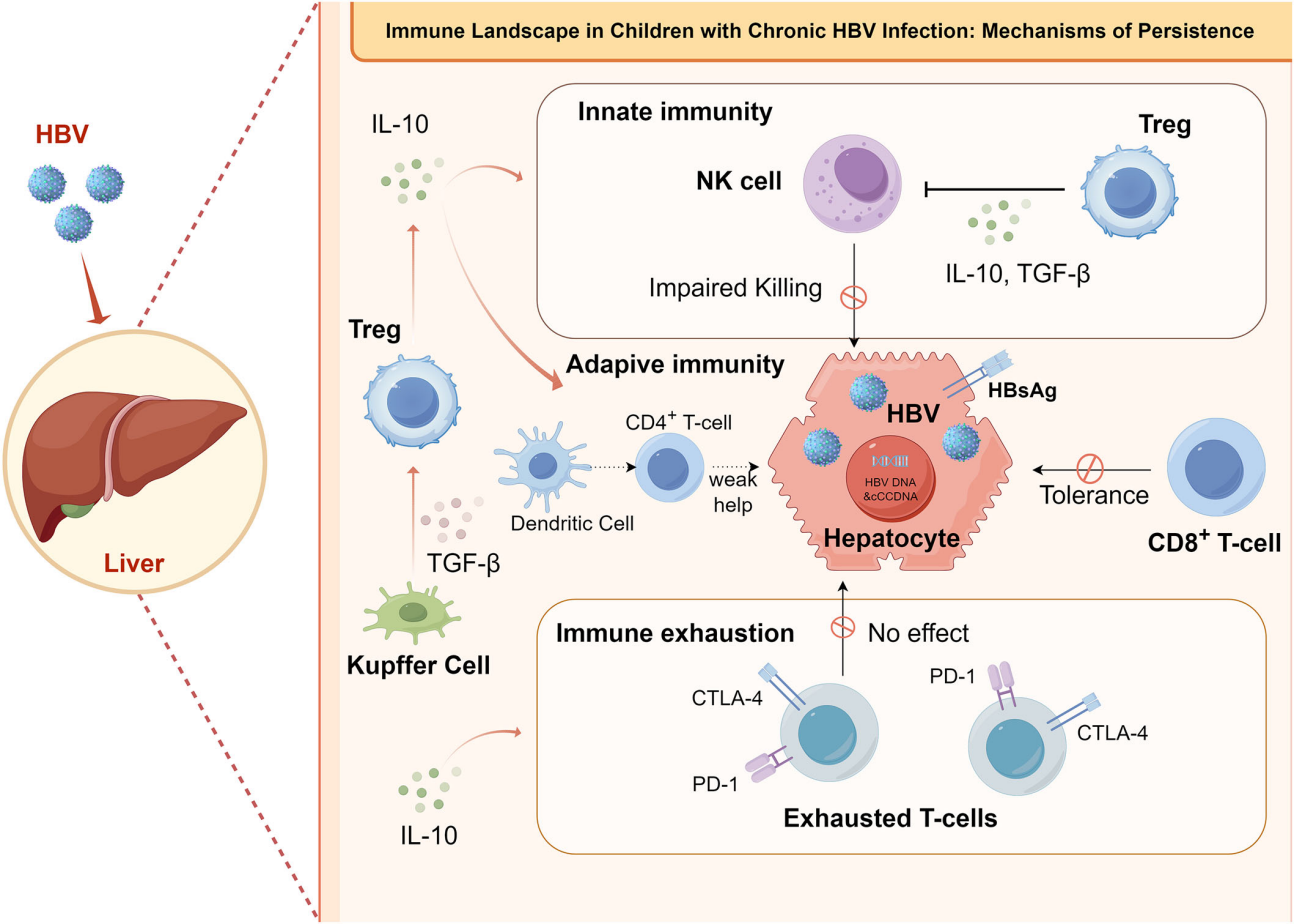
Immune landscape and mechanisms of viral persistence in chronic HBV infection in children. This figure illustrates the key immune mechanisms underlying viral persistence in children with chronic HBV infection, highlighting impairments across innate, adaptive, and effector immune pathways. In the innate immune compartment, NK-cells cytotoxicity is impaired, and regulatory T cells (Tregs) further suppress antiviral activity via the secretion of inhibitory cytokines such as IL-10 and TGF-β. Within adaptive immunity, dendritic cells exhibit functional defects, leading to inadequate helper signals from CD4^+^ T cells for CD8^+^ T cells, while abundant viral antigen (HBsAg) on infected hepatocytes induces CD8^+^ T-cell tolerance. Additionally, chronic viral stimulation drives the upregulation of inhibitory receptors (PD-1, CTLA-4) on T cells, resulting in T-cell exhaustion and loss of effector function, which is exacerbated by IL-10 secretion from Kupffer cells. Collectively, these immunosuppressive mechanisms create a hepatic microenvironment that favors HBV persistence.

CHB in children presents a distinct immunological landscape compared to adults, largely due to the developing immune system in pediatric populations (17, 18). Comprehending the immune response mechanisms to HBV infection in pediatric populations is crucial for the advancement of therapeutic strategies and the identification of novel treatment targets (Refer to **Figure 2** for a detailed depiction of the specific immune response).

Interferon exerts its anti HBV effect mainly through two pathways: direct viral suppression and indirect immune regulation. For children with immature immune systems, immune regulation is its core mechanism of action (19). Polyethylene glycol interferon is a long-acting interferon modified with polyethylene glycol, which has an extended half-life and longer biological activity. It overcomes the shortcomings of frequent administration and large fluctuations in blood drug concentration of ordinary interferons and is more suitable for pediatric clinical applications (20). Although PEG-IFN cannot directly degrade cccDNA, which serves as a sustained viral replication library in liver cells, it can inhibit the transcription and replication of cccDNA by upregulating the expression of interferon stimulated genes (ISGs) in liver cells. Interferon stimulated genes such as protein kinase R (PKR) and 2 ‘-5’ oligoadenylate synthase (2 ‘-5’ OAS) can effectively inhibit the translation of HBV protein, degrade viral RNA, and then reduce the expression level of hepatitis B surface antigen (HBsAg) and e antigen (HBeAg) (21). PEG-IFN can activate dendritic cells (DCs), natural killer (NK) cells, and natural killer T cells (NKT) in children’s bodies, enhance the anti HBV activity of the innate immune system, promote dendritic cell maturation and effective presentation of viral antigens, and enhance the cytotoxic effect of NK cells on HBV infected liver cells. At the level of adaptive immunity, PEG-IFN can stimulate the proliferation and differentiation of HBV specific CD4^+^ helper T cells and CD8^+^ cytotoxic T lymphocytes (CTLs), reverse the immune tolerance of children’s immune system to HBV, initiate the body’s immune-mediated specific virus clearance process, and achieve long-term virus suppression (22).

In children, the immune response to HBV includes both innate and adaptive immunity, prominently involving T-cells and NK cells (23). The innate immune system serves as the initial defense, with NK cells crucially targeting HBV-infected cells early in infection (24, 25). However, in chronic HBV cases, the function of these innate cells is often impaired, preventing an effective immune response and allowing the virus to persist in the liver (26). The adaptive immune response, involving HBV-specific T-cells, is crucial in chronic infection progression (27). In children, HBV-specific CD8^+^ CTLs are often less effective at clearing infected hepatocytes due to early-life immune tolerance (28, 29). Additionally, CD4^+^ T-helper cells, which aid CD8^+^ T-cells, may be compromised, resulting in a weak immune response against HBV (30, 31).

A recent study by Tian et al. found that defects in HBV-specific CD8^+^ T cells in neonatal mice infected at birth were linked to inhibitory actions by liver macrophages, increasing the risk of vertical transmission and chronic infection (32). Immune exhaustion significantly contributes to chronic HBV in children, as exhausted T-cells with inhibitory receptors like PD-1 and CTLA-4 hinder effective responses against the virus (18, 33, 34). In adults, the immune response is typically more active, though it can lead to liver damage.

The hepatic microenvironment in pediatric patients with chronic HBV infection presents additional complexities to the immune response (35). The liver, as a distinct organ, inherently favors immune tolerance, a mechanism crucial for averting hyperactive immune reactions that may result in autoimmune pathologies (36). However, this same propensity for tolerance fosters conditions that facilitate viral persistence (36, 37). The abundance of regulatory T-cells within the hepatic milieu, which function to suppress immune activity, is likely instrumental in enabling HBV to circumvent immune detection, thereby perpetuating the chronicity of the infection (38–40). Furthermore, hepatic replication of the HBV is associated with persistent immune activation and inflammatory processes, which can ultimately result in fibrosis (41, 42). Over an extended period, this sustained immune response has the potential to inflict hepatic damage and promote fibrotic changes, thereby elevating the risk of cirrhosis and HCC in both pediatric and adult populations (43).

## 3 Interferon therapy in CHB in children

### 3.1 Mechanism of interferon therapy for CHB in children

IFN therapy, especially PEG-IFN, has been a cornerstone of treatment for CHB, even in pediatric populations (13). While nucleos(t)ide analogues (NAs) such as entecavir and tenofovir are widely used due to their potent antiviral activity and favorable safety profile, interferon offers unique advantages (11, 44, 45). Interferon not only suppresses viral replication but also modulates the immune system, enhancing the host’s ability to control HBV infection (46, 47). In children, the immune modulation induced by interferon can play a crucial role in achieving sustained virological response (SVR) and potentially clearing the virus (16, 48).

IFN mediates its therapeutic effects through a variety of mechanisms, encompassing both direct antiviral actions and immune-modulatory functions (49). As a cytokine, IFN directly suppresses HBV replication by binding to specific receptors on hepatocytes, thereby initiating a cascade of intracellular signaling pathways (49–51). These pathways culminate in the expression of antiviral proteins, notably MX1, OAS, and PKR, which collectively inhibit viral replication and bolster the host cell’s antiviral defense mechanisms (52, 53). Furthermore, interferon augments the capacity of T-cells and NK cells to identify and eliminate HBV-infected hepatocytes (54, 55). This immune activation is pivotal for the sustained control of the virus, especially when used in conjunction with other antiviral therapies (48). Furthermore, IFN has a profound impact on the immune system by modulating the balance between immune tolerance and immune activation (56). It can shift the immune response from a tolerogenic state, which is typically observed in chronic HBV infections, to a more active and potentially virus-eradicating response (57). This immune modulation can reduce the immune suppression typically seen in chronic infections, allowing for more effective viral clearance (58). In the pediatric population, this immune-enhancing property of interferon is particularly important, as it helps overcome the typically weaker immune response seen in children with chronic HBV (2, 48).

The administration of IFN in pediatric populations poses considerable challenges, encompassing issues related to treatment efficacy, adverse effects, and patient adherence (13). Specifically, PEG-IFN necessitates weekly subcutaneous injections, which can be onerous for both children and their families (59). Furthermore, IFN therapy is associated with adverse effects such as flu-like symptoms, cytopenia, and hepatotoxicity, which may compromise treatment adherence and result in premature discontinuation (59, 60). Nonetheless, despite these obstacles, interferon therapy continues to be an important component in the management of CHB, particularly when used in conjunction with other therapeutic strategies (59, 60).

Combination Therapy has emerged as a promising approach to enhance the efficacy of IFN in treating pediatric CHB (45, 61). Combining IFN with NAs or other immunomodulatory agents may address the limitations of monotherapy, such as the risk of resistance and the incomplete immune activation seen with NAs alone (62). Recent studies have shown that the combination of interferon with NAs can provide a dual benefit: antiviral suppression through NAs and immune system modulation through interferon (63). This combination approach has shown promise in increasing the likelihood of achieving a sustained virological response and even viral clearance in some pediatric patients (64).

Polyethylene glycol interferon alpha-2a/alpha-2b is the preferred interferon preparation for pediatric CHB treatment. Clinical medication should be stratified according to the age and body weight of children. The specific dosage and course of treatment are: 3–6 year old children, administered according to body weight, PEG-IFNα-2b 6μg/kg, PEG-IFN alpha-2a 10000 IU/kg, subcutaneous injection once a week; 7-12-year-old children, PEG-IFN alpha-2b 5 μg/kg (maximum dose not exceeding 180 μg), or PEG-IFN alpha-2a 8000–10000 IU/kg, subcutaneous injection once a week; Adolescents aged 12 and above should receive subcutaneous injections of PEG-IFN alpha-2b 180 μg/time or PEG-IFN alpha-2a 180 μg/time once a week, following the adult dosage. The conventional treatment course is 48 weeks. If there is no significant decrease in HBsAg (decrease<1 log10 IU/mL) and HBV DNA does not turn negative after 24 weeks of treatment, it indicates poor treatment response and may consider discontinuing the medication or adjusting the treatment plan. Recently, multiple clinical studies have explored the efficacy of IFN - based treatment regimens in children with CHB. A study involving 76 children aged 3–18 years old treated with pegylated interferon alpha-2b combined with entecavir showed that 31.6% of patients achieved HBsAg clearance (65). Another controlled study was conducted on 70 children aged 3–18 years who were initially treated, comparing the efficacy of pegylated interferon alpha-2a and entecavir monotherapy. It was found that at 48 weeks of treatment, the HBsAg serological response (SR) rate and HBeAg serological response rate in the pegylated interferon group were significantly higher than those in the entecavir monotherapy group (66). In addition, a comprehensive analysis of multiple studies involving 1121 children with CHB showed that the combination therapy of pegylated interferon and NAs drugs was superior to pegylated interferon monotherapy or NAs monotherapy in terms of HBV DNA undetectable rate, HBeAg clearance rate, HBeAg seroconversion rate, and alanine aminotransferase (ALT) normalization rate (67).

In addition, the adverse reactions of interferon treatment have a clear incidence rate, among which influenza like symptoms (fever, fatigue, muscle soreness) have the highest incidence rate of over 90%, mostly appearing in the first 4 weeks of treatment and gradually improving with the progress of treatment (68). The incidence of leukopenia is about 30%–40%, with leukopenia (20%–30%) and thrombocytopenia (10%–15%) being the main causes, mostly in grades 1–2. The incidence of severe leukopenia in grades 3–4 is less than 5% (69). In addition, the incidence of rash and gastrointestinal reactions (nausea, decreased appetite) is about 15%–20%. These adverse reactions may affect treatment compliance and lead to premature discontinuation of medication, and clinical monitoring and symptomatic treatment are needed in a timely manner (70–72).

### 3.2 Effects of IFN therapy on children’s growth and development

The impact of PEG-IFN treatment on the growth and development of children is a core safety issue of clinical concern. Existing clinical data shows that short-term (within 48 weeks) PEG-IFN treatment has no sustained negative effects on the growth of height and weight in children aged 3–18. Only a few children experience a slowdown in weight growth in the first 3 months of treatment, and can recover to the normal growth curve of children of the same age after discontinuation (73). At the endocrine level, approximately 5% of pediatric patients experience transient thyroid dysfunction (hyperthyroidism/hypothyroidism) during treatment, with subclinical hypothyroidism being the main cause. More than 80% of patients recover normal thyroid function within 6 months after discontinuation of medication, and no permanent endocrine damage is found (74). In addition, there is no clear research confirming that PEG-IFN treatment affects other growth and development indicators such as bone development and sexual development in children. However, there is still a lack of relevant data on long-term (over 1 year) treatment, and further follow-up studies are needed.

### 3.3 Pretreatment biomarkers for predicting the therapeutic effect of IFN in children with hepatitis B

In clinical practice, the response effect of children to PEG-IFN treatment can be predicted by the biomarkers before treatment. The core effective markers include: ① the level of HBsAg (75): for children with HBsAg<1000 IU/mL before treatment, the sustained virological response rate after 48 weeks of PEG-IFN treatment is significantly higher than that of those with high titer; ② The level of HBeAg and serum ALT (59, 76, 77): The children with low HBeAg titer (<100 S/CO) and continuous increase of ALT (2–5 times the upper limit of normal value) have better immune activation status and higher treatment response rate; The combined detection of the above markers can improve the accuracy of efficacy prediction and provide a basis for clinical screening of suitable children for treatment.

## 4 Challenges and limitations

IFN therapy, especially PEG-IFN, has always been the cornerstone of treating CHB in children, but its application faces significant challenges. One of the most prominent issues is the limited effectiveness of treatment. In addition, side effects and tolerability remain major issues. PEG-IFN is associated with flu like symptoms, decreased cell count, and liver toxicity, which can have a negative impact on treatment compliance (60). These adverse reactions are particularly heavy for children, making it difficult for them to complete the entire treatment process. Another challenge is immune tolerance and fatigue (13, 14). Chronic HBV infection in children leads to the development of immune tolerance, which limits the immune system’s ability to effectively respond to the virus (78). In addition, the depletion of effector T cells over time further hinders the antiviral immune response, leading to the persistence of the virus (79). Despite these challenges, there is still a great opportunity to improve interferon therapy for children with CHB. The combination therapy involving PEG-IFN and NA has shown promise, as they combine the antiviral properties of NAs with the immunomodulatory effects of IFN (62, 80). This dual approach can enhance antiviral response and improve efficacy, but it may also increase the risk of adverse reactions. In addition, personalized treatment strategies have great potential in optimizing IFN therapy (81). By combining genetic and immune biomarkers, clinicians can better tailor treatment plans for individual patients, improving efficacy and safety (82). Another opportunity lies in the advancement of drug formulations and delivery systems, such as the development of long-acting PEG-IFN or new delivery methods, which can improve patient compliance, reduce injection frequency, and make treatment for children more feasible.

In addition to overcoming these challenges, future research should also focus on several key areas. Immune tolerance is the main obstacle to effective IFN therapy for CHB in children. One solution is to combine IFN with immune modulators (such as anti-PD-1/PD-L1 inhibitors) to enhance immune activation and overcome tolerance (83). This combination method may activate exhausted T cells and enhance immune response, thereby promoting more effective virus clearance. Finally, personalized medicine driven by biomarkers is crucial for selecting appropriate IFN therapy patients to improve treatment outcomes (60). Identifying biomarkers of treatment response will help target IFN therapy, ensuring that children who are most likely to benefit from treatment receive treatment while minimizing unnecessary exposure to others.

## 5 Future directions

With the continuous development of the treatment field for CHB, some technological advances and innovative strategies provide promising ways to improve IFN therapy in children. The development of long-acting IFN preparations, such as PEG-IFN with sustained release properties, can significantly improve patient compliance by reducing injection frequency. In addition, combining IFN with immune checkpoint inhibitors or immunomodulators may further activate immune responses and overcome common immune tolerance in pediatric populations (84). These advances are expected to reduce side effects, improve treatment efficacy, and may bring functional cure to some children with CHB. In addition, exploring gene editing technologies such as CRISPR that directly target HBV DNA can provide a clearer solution for chronic infections, although this approach is still in its early stages of development (85). Looking ahead, further research is urgently needed to optimize IFN therapy for children with CHB. Research should focus on personalized treatment plans tailored to the immune characteristics and genetic background of pediatric patients. Biomarkers that predict treatment response play a crucial role in determining which children will benefit the most from IFN therapy, enabling more targeted interventions and reducing unnecessary treatment (86). In addition, clinical trials involving the combination of IFN with novel antiviral drugs or immunomodulatory therapies should be prioritized to evaluate their potential for achieving better results. Research should also explore the long-term safety and efficacy of interferon in pediatric populations, particularly its impact on growth, development, and autoimmune risk (13, 87).

From a policy perspective, it is necessary to advocate for broader insurance coverage and updated guidelines to ensure that all children in need have access to IFN treatment. The government and medical institutions should strive to expand the medical insurance coverage of IFN therapy, as cost remains a significant obstacle to treatment in many regions. In addition, updated clinical guidelines are crucial for reflecting the latest research findings and incorporating combination therapies and personalized approaches. Immediate action is needed to ensure that children with CHB receive the most effective treatment. By prioritizing research, improving accessibility, and adjusting clinical practice, we can significantly reduce the burden of this disease. Early intervention and innovation can ultimately avoid the long-term consequences of chronic HBV infection, such as cirrhosis and cancer, in future generations (88).

## 6 Discussion

From this perspective, we emphasize the crucial role of IFN therapy in the management of CHB in pediatric patients, highlighting its immunological advantages and inherent challenges in its application. Although IFN remains a fundamental component in the treatment of pediatric CHB, its limitations in efficacy, adverse reactions, and the complexity of pediatric immune tolerance require further research. The potential of combining IFN with other antiviral drugs or immunomodulatory therapies provides a promising avenue for improving treatment outcomes and addressing existing challenges. The clinical translation potential of these insights is enormous. By focusing on personalized treatment, improving drug formulations, and addressing treatment resistance, we can move towards more effective and sustainable interventions. In addition, interdisciplinary collaboration between immunologists, hepatologists, and pediatricians is crucial for optimizing treatment strategies for CHB. Rapid progress in these areas can significantly improve the quality of life of children affected by HBV, ultimately reducing the long-term consequences of chronic infections.

## 7 Conclusion

This article systematically analyzed the mechanism of action, clinical application status, challenges, and future directions of IFN (especially PEG-IFN) in the treatment of CHB in children from an immunological perspective. Combined with clinical quantitative data and biomarker research, the core value and application norms of PEG-IFN in the treatment of pediatric CHB were clarified. PEG-IFN is recommended as the first-line treatment for CHB in children aged 3 and above. Medication should follow the age/body weight stratified dose principle (6 μg/kg (alpha-2b)/10000 IU/kg (alpha-2a) for 3–6 years old, 5 μg/kg (alpha-2b)/8000–10000 IU/kg (alpha-2a) for 7–12 years old, and 180 μg/time for adults aged 12 and above). Subcutaneous injection should be given once a week, with a routine course of 48 weeks. Before treatment, suitable children can be screened for HBsAg, HBeAg, ALT, and ISGs expression levels. Adverse reaction monitoring and symptomatic treatment should be strengthened during treatment.

## Data Availability

The original contributions presented in the study are included in the article/supplementary material. Further inquiries can be directed to the corresponding author.
